# Supplementary figures and images for: The Regulatory Axis of PD-L1 Isoform 2/TNF/T Cell Proliferation Is Required for the Canonical Immune-Suppressive Effects of PD-L1 Isoform 1 in Liver Cancer
Source: Int J Mol Sci. 2023 Mar 28;24(7):6314. doi: 10.3390/ijms24076314 (PMC10094247; doi:10.3390/ijms24076314)

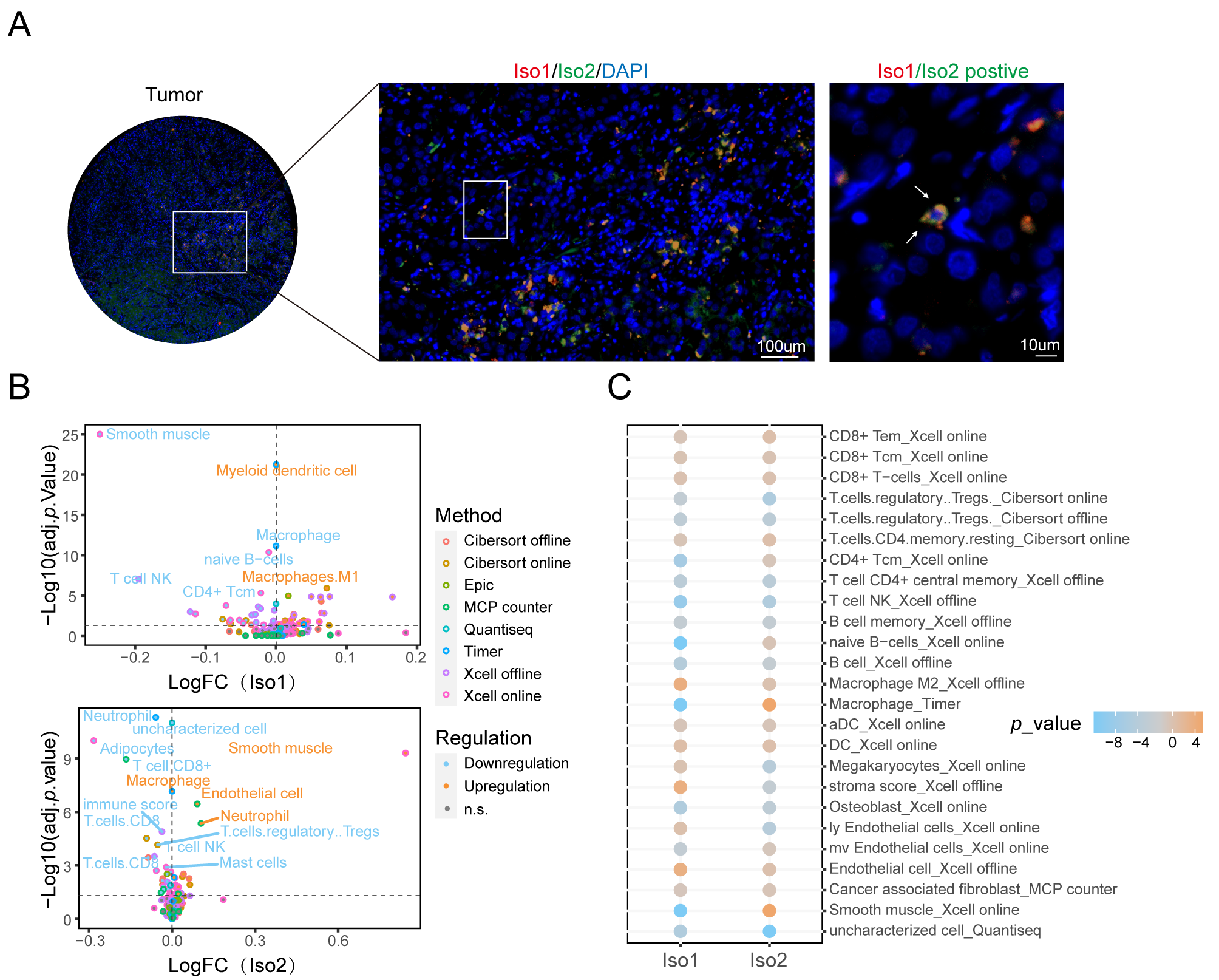

Supplement: Supplementary file 1 [file ijms-24-06314-s001.zip › Figure S1.tif]

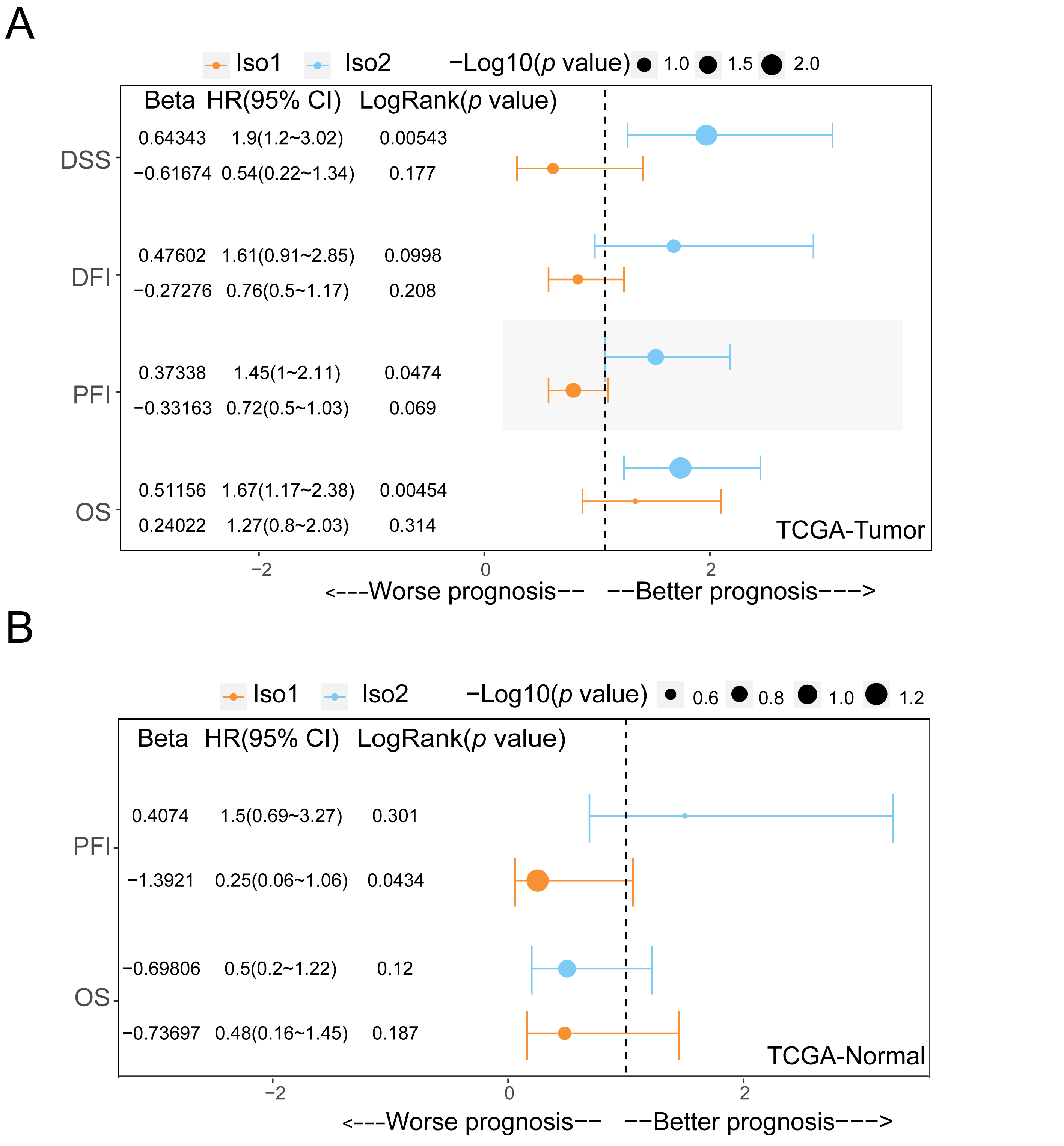

Supplement: Supplementary file 1 [file ijms-24-06314-s001.zip › Figure S2.tif]

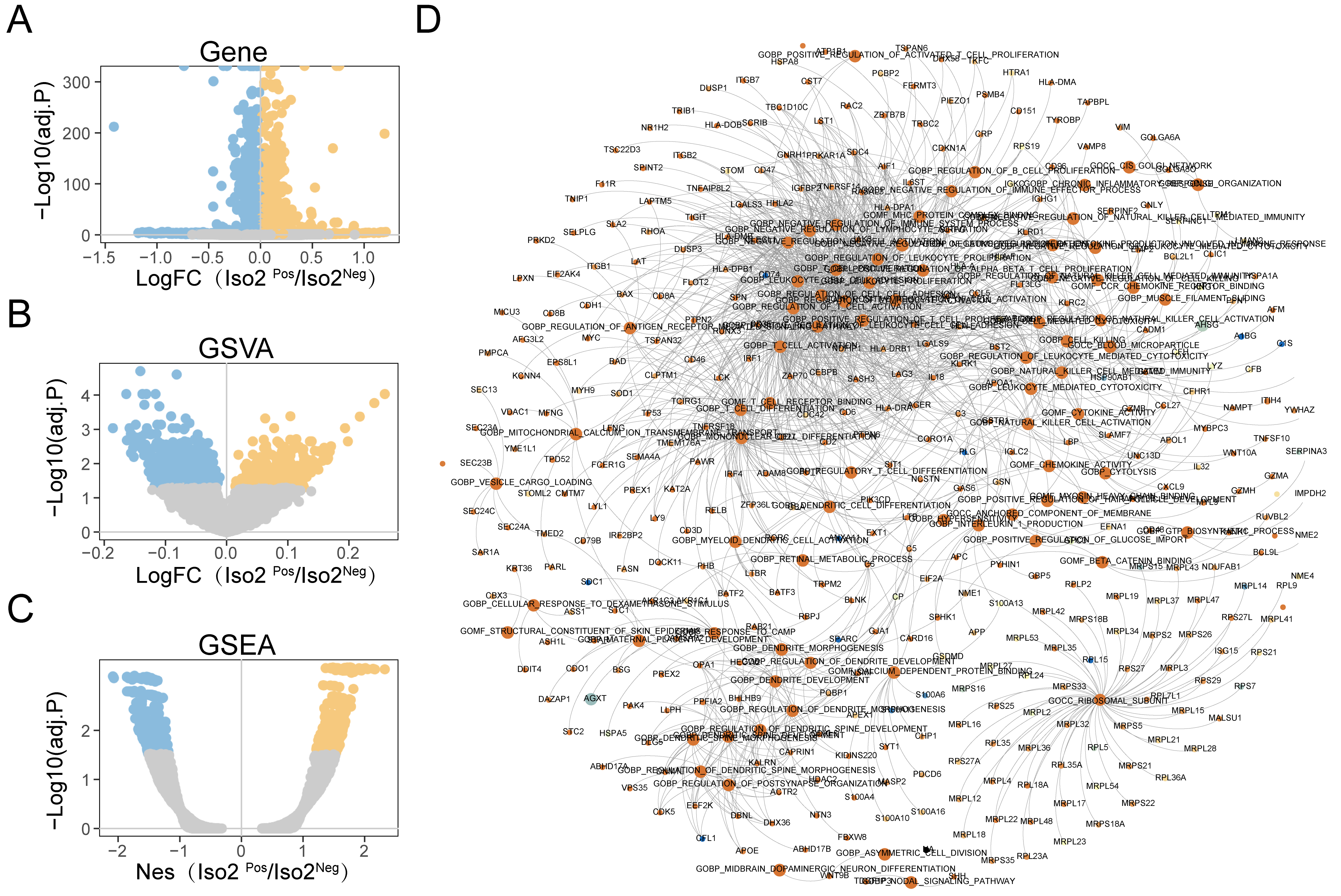

Supplement: Supplementary file 1 [file ijms-24-06314-s001.zip › Figure S3.tif]
